# Recurrent *CYP2C19* deletion allele is associated with triple-negative breast cancer

**DOI:** 10.1186/1471-2407-14-902

**Published:** 2014-12-02

**Authors:** Anna Tervasmäki, Robert Winqvist, Arja Jukkola-Vuorinen, Katri Pylkäs

**Affiliations:** Department of Clinical Chemistry and Biocenter Oulu, Laboratory of Cancer Genetics and Tumor Biology, University of Oulu, P.O. Box 5000, Oulu, FI 90014 Finland; Laboratory of Cancer Genetics and Tumor Biology, Northern Finland Laboratory Centre, NordLab, Oulu, Finland; Department of Oncology, University of Oulu, Oulu University Hospital, P.O. Box 5000, Oulu, FI 90014 Finland

**Keywords:** Copy number variation, CYP2C19, Triple-negative breast cancer

## Abstract

**Background:**

Using a genome-wide approach, we have previously observed an increase in the frequency of rare copy number variants (CNVs) in familial and early-onset breast cancer cases when compared to controls. Moreover, the biological networks of the CNV disrupted genes differed between the two groups. Here, six of the previously observed CNVs were selected for further investigation. Four of these were singletons and disturbed the following genes: *DCLRE1C*, *CASP3*, *DAB2IP* and *ITGA9*, encoding proteins that are part of the *TP53* and β-estradiol centered network. The two others were recurrent alleles and disrupted *CDH19* and *CYP2C19* genes. Of these, *CDH19* encodes a cadherin functioning as a cell-cell adhesion receptor and *CYP2C19* a CYP450 enzyme with a major function in estrogen catabolism.

**Methods:**

The exact breakpoints of the six previously observed CNV deletion alleles were defined by using qPCR, nested PCR and sequencing. The prevalence of these CNVs was investigated in 842 Northern Finnish breast cancer cases, unselected for family history of cancer and age at disease onset, as well as in 497 healthy female controls by using multiplex PCR. Also the association of the relatively common *CDH19* and *CYP2C19* deletion alleles with different clinical parameters was studied.

**Results:**

No significant differences in the carrier frequencies between cases and controls were found for any of the studied CNVs. However, the deletion in *CYP2C19* showed a significant association with triple-negative breast cancer (*p* = 0.021).

**Conclusion:**

Our results indicate that inherited changes in *CYP2C19* gene participating in estrogen catabolism have an influence on the molecular subtype of breast cancer.

**Electronic supplementary material:**

The online version of this article (doi:10.1186/1471-2407-14-902) contains supplementary material, which is available to authorized users.

## Background

Copy number variants (CNVs) are genomic microduplications or microdeletions which can affect gene function and predispose to various diseases [1], including breast cancer. Although no evidence for the association of common CNVs with breast cancer susceptibility has been reported [2], recent genome-wide studies suggest that rare CNVs represent an alternative source of genetic variation influencing hereditary breast cancer risk [3, 4]. In our previous study, we observed a consistent increase in the frequency of rare CNVs in familial and early-onset breast cancer cases when compared to controls. Furthermore, the biological networks of the disrupted genes differed between the two groups: the disrupted genes in breast cancer cases were shown to be closely related to estrogen signalling and *TP53* centered tumor suppressor network [4].

Based on their biological functions and recurrence, two of the previously identified deletion alleles disrupting *CYP2C19* and *CDH19* genes, respectively, were hypothesized to play a role in breast cancer predisposition also in the general population. Of these, *CYP2C19* encodes a CYP450 enzyme with a major function in estrogen catabolism: it catalyzes 17 β-hydroxy dehydrogenation and 16 α-hydroxylation of estradiol [5, 6]. CYP2C19 has also been reported to participate in tamoxifen metabolism during breast cancer treatment, as it contributes in tamoxifen 4-hydroxylation [7]. The decreased activity of CYP2C19 through haploinsufficiency might be related to an increase in breast cancer risk, potentially through life-long increased estrogen levels [4]. In contrast, *CDH19* encodes a cadherin, which is a cell-cell adhesion receptor establishing and maintaining intercellular connections. Loss of function of cadherins may be connected to cancer formation [8]. In our previous study, the *CYP2C19* deletion allele was found twice as frequent in familial breast cancer cases (5.8%) as in controls (2.3%), whereas *CDH19* was observed once in both familial (1.0%) and control cohorts (0.8%) [4], implicating the need for a larger dataset for the evaluation of their disease relatedness. Besides the breast cancer risk itself, both changes could also have an effect on tumor biology.

Although a majority of the previously observed CNV alleles, which disrupted genes from the *TP53* and β-estradiol centered network, were singletons [4], some could represent founder mutations typical for the Finnish population. Thus, based on their biological functions, four singleton deletion alleles disrupting *CASP3, DAB2IP*, *DCLRE1C* and *ITGA9* genes, respectively, were included in the study. Of these CASP3 functions in apoptosis, failure of which can lead to cancer [9]. *DAB2IP* encodes a member of the RAS GTPase-activating gene family and has been reported to act as a tumor suppressor: its inactivation by promoter methylation occurs in several malignancies, including prostate and breast cancer [10]. DCLRE1C operates in the DNA double-strand break repair pathway, defect of which has been strongly associated with breast cancer predisposition [11], and *ITGA9* encodes α-integrin, which participates in the control of cell division, differentiation and migration [12–14]. The chromosomal region harboring *ITGA9* has been reported to be deleted in several epithelial malignancies, including breast carcinoma [15].

Here we have defined the exact breakpoints of six previously identified deletion alleles disrupting the *CYP2C19, CDH19, CASP3, DCLRE1C, DAB2IP* and *ITGA9* genes, respectively, and evaluated their association with breast cancer risk and disease subtype using a Northern Finnish case–control cohort. As a result, we provide evidence suggestive of the *CYP2C19* deletion allele being associated particularly with susceptibility to triple-negative breast cancer.

## Methods

### Subjects

Patient cohort consisted of 842 Northern Finnish breast cancer cases diagnosed at the Oulu University Hospital between the years 2000 and 2011. All cases were unselected for a family history of the disease and age at disease onset. The median age at diagnosis for cases was 57 years (variation 28–92 years). 497 geographically matched anonymous cancer-free female Northern Finnish Red-Cross blood donors (median age at monitoring was 42 years, variation 18–66 years) were used as controls. Control samples were provided by Finnish Red Cross Blood Service, with the information only about their gender, age and place of blood donation. Controls have given their informed consent to use part of their sample for research purposes at the time of donation. The genomic DNA of cases and controls was extracted from blood samples using either the standard phenol-chloroform method, Puregene D-50 K purification kit (Gentra, Minneapolis, MN, USA), or UltraClean Blood DNA Isolation Kit (MoBio, Carlsbad, CA, USA)

For 551 of the breast cancer cases we had access to the clinical parameters obtained from the pathology reports. These included tumor histology, grade, size, nodal status, distant metastases, estrogen receptor (ER), progesterone receptor (PR), HER2 and Ki-67 status. For ER and PR, positive staining was defined as nuclear immunostaining in 1 to 10% (weak), 10 to 50% (moderate), or >50% (strong) of the tumor cells, whereas negative indicated no staining. HER2 expression was studied by means of immunohistochemistry (positivity defined as weak, moderate or strong levels of staining and negativity completely negative staining) and chromogenic *in situ* hybridization (CISH). Cut-off values used for Ki-67 were negative (0), weak (1), moderate (2) and strong (3). ER, PR and HER2 status were used as surrogate markers to divide the tumors further into luminalA, luminalB, HER2 type and triple-negative subtypes [16, 17]. LuminalA was defined as positive ER or PR expression and no HER2 overexpression, luminalB had positive ER or PR and HER2 overexpression, HER2 type as negative ER and PR but with HER2 overexpression, and triple-negative as negative for all three markers.

All specimens and clinical information were collected with the informed consent of the patients. This study was approved by the Ethical Board of the Oulu University Hospital Health Care District and the Finnish Ministry of Social Affairs and Health.

### Genotyping of the deletion alleles

The exact breakpoints of *CYP2C19, CDH19, CASP3, DCLRE1C, DAB2IP* and *ITGA9* genes disrupting deletion CNVs were defined by performing qPCR (BioRad CFX96, BioRad, Hercules, CA, USA) and nested PCR (GeneAmp High Fidelity PCR System, Applied Biosystems, Foster City, CA, USA) with the primers surrounding the breakpoint coordinates received from Illumina HumanOmni1-Quad BeadChips, analyzed with GenomeStudio Genotyping module (Illumina Inc., San Diego, CA, USA) and Nexus Copy Number Discovery Edition 5.1 software (BioDiscovery Inc. El Segundo, CA, USA) [4]. The breakpoints were verified by direct sequencing (ABI3130xl Genetic Analyzer, Applied Biosystems, Foster City, CA, USA). Nested allele-specific PCR for the detection of the deletion alleles was designed in a multiplex format, containing control primers to monitor PCR success. Deletion CNV containing samples were used as positive controls. Primers to amplify the deletion alleles are presented in Additional file 1: Table S1. The obtained PCR amplicons were analyzed with Bioanalyzer (Agilent Technologies, Waldbronn, Germany). All the observed deletion carrier samples were verified by direct sequencing. The heterozygosity of the *CYP2C19* and *CDH19* deletion alleles was confirmed by second, wild type allele specific PCR. For *CYP2C19,* the same forward primer as in multiplex reaction was used, reverse (ACTTGACGATGGAGGGTGAA) resided on genomic region present only in wild type allele. For *CDH19,* the reverse primer was the same as in multiplex reaction, whereas the forward primer (TCTGAATCTGGTGAGGGAACA) was wild type specific.

### Genotyping for other *CYP2C19*metabolizer alleles in *CYP2C19*deletion carriers

The status of the remaining *CYP2C19* allele both in carriers with breast cancer and those remaining healthy was investigated by genotyping for the three literature described metabolizer types: CYP2C19*2 (c.681G > A, rs4244285, poor metabolizer), CYP2C19*3 (c.636G > A, rs57081121, poor metabolizer) and CYP2C19*17 (-806C > T, rs12248560, ultra-rapid metabolizer) [18–21]. Genotyping was performed with direct sequencing (ABI3130xl Genetic Analyzer) using primers in Additional file 1: Table S2 [22–24].

### Statistical analysis

Statistical analyses were performed with IBM SPSS Statistics 20 (IBM Corporation, Armonk, NY, USA). *P*-values for comparisons between cases and controls and for the evaluation of the differences in tumor characteristics were obtained using Pearson’s chi-squared or Fisher’s exact test. Fisher’s exact test was used if any of the crosstab cells had expected count less than 5. *P-*values were not corrected for multiple testing in order not to eliminate potentially significant findings obtained with small number of CNV carriers. All *p*-values were two-sided.

## Results

The exact coordinates for the deletion alleles corresponded well to that received from microarrays [4], except for *DAB2IP*, for which the deletion was significantly larger than originally predicted (Additional file 1: Table S3). The sequencing verified genomic coordinates of the deletions and the carrier frequencies in the analyzed cohorts are presented in Table 1. When performing case–control comparisons, no additional *CASP3, DCLRE1C* or *DAB2IP* deletion allele carriers were observed, indicating that these alleles are either truly singletons or extremely rare. In contrast, *ITGA9* deletion allele carriers were observed once in cases and once in controls, leaving its potential role in breast cancer predisposition uncertain. Of the two recurrent alleles, the *CDH19* deletion showed higher frequency in cases (12/842, 1.4%) than in controls (3/497, 0.6%), but because of its rarity the difference remained below the level of statistical significance (*p* = 0.168). The *CYP2C19* deletion carrier frequency was only marginally higher in the breast cancer cases (31/842, 3.7%) when compared to the controls (17/497, 3.4%), being relatively high in both groups (sequence of the *CYP2C19* deletion breakpoints and its surrounding area is presented in Additional file 2: Figure S1). All *CDH19* and *CYP2C19* mutation positive individuals were verified to be heterozygotes.

**Table 1:** **Genomic coordinates, sizes and carrier frequencies of the studied deletion alleles**

Disrupted gene	Location of deletion	Deletion size	Carrier frequency, n/N (%)		***P***-value	OR (95% CI)
			Breast cancer cases controls			
*DCLRE1C*	Chr10: 14,983,925–15,065,676	82 kb	0/842 (-)	0/497 (-)	NA	NA
*CASP3*	Chr4: 185,506,876–185,841,468	335 kb	0/842 (-)	0/497 (-)	NA	NA
*DAB2IP*	Chr9: 124,201,774–124,361,084	159 kb	0/842 (-)	0/497 (-)	NA	NA
*ITGA9*	Chr3: 37,750,166–37,810,925	61 kb	1/842 (0.1%)	1/497 (0.2%)	1.000	0.590 (0.037 – 9.450)
*CDH19*	Chr18: 64,082,045–64,335,669	254 kb	12/842 (1.4%)	3/497 (0.6%)	0.168	2.381 (0.669 – 8.478)
*CYP2C19*	Chr10: 96,497,324–96,559,110	62 kb	31/842 (3.7%)	17/497 (3.4%)	0.804	1.079 (0.591 – 1.971)

NA = not available, OR = odds ratio, CI = confidence interval. Genomic coordinates according to human genome assembly 19 (February 2009).

The case–control comparisons were followed by case-case analysis for the differences in clinical parameters between CNV carriers and non-carriers. The tumor characteristics of *CDH19* deletion carriers did not significantly differ from non-carrier cases, although the carrier tumors tended to be more frequently of higher grade (7/12, 58.3%, of the tumors categorized as grade 3) than the non-carrier tumors (197/522, 37.7%) (Additional file 1: Table S4). In contrast, for *CYP2C19* the tumors of deletion carriers showed association with negative ER (*p* = 0.048) and PR status (*p* = 0.078), the latter, however, remaining slightly below the level of statistical significance. HER2 negativity was at similar level as in wild type tumors (Table 2). When combining all three parameters, *CYP2C19* deletion carriers were at significantly higher risk for developing triple-negative (ER/PR/HER2 negative) tumors than non-carriers (*p* = 0.021, OR 2.83; 95% CI 1.20-6.66). Altogether, these results indicate that the tumor characteristics of *CYP2C19* deletion carriers are related to estrogen responsiveness. As the known biological function of CYP2C19 is related to estrogen catabolism, and as the studied deletion allele is expected to be a null allele, we further defined the status of the remaining *CYP2C19* allele in relation to literature described metabolizer genotypes both in carriers with breast cancer and those remaining healthy. No CYP2C19*3 poor metabolizers were identified, whereas four CYP2C19*2 poor metabolizers were identified in patients (4/31, 12.9%) and one in controls (1/17, 5.9%). There was no difference in the frequency of CYP2C19*17 ultra-rapid metabolizer genotypes (7/31, 22.6% in patients vs. 3/17, 17.6% in healthy controls), indicating that the disease risk of individual with *CYP2C19* deletion was not significantly affected by the metabolizer status of the remaining allele. Furthermore, in a majority (7/10) of the deletion carriers with ER negative cancer the second allele was found to be wild type, whereas one (1/10) had a poor-metabolizer CYP2C19*2 allele and the rest (2/10) an ultra-rapid metabolizer CYP2C19*17 allele.

**Table 2:** **Tumor characteristics of** ***CYP2C19*** **deletion allele carriers compared with the tumors of non-carrier unselected breast cancer cases**

Category	***CYP2C19***deletion	%	Wild type	%	***P***-value ^a^	OR	95% CI
T							
1	18	62.0%	304	58.3%			
2	10	34.5%	188	36.1%	0.692	1.17	0.54-2.52
3	1	3.5%	16	3.1%	1 vs. 2,3,4		
4	0	0%	13	2.5%			
N							
Neg	20	64.5%	293	56.0%	0.354	1.43	0.67-3.04
Pos	11	35.5%	230	44.0%			
M							
Neg	31	100%	500	95.6%	0.632	NA	
Pos	0		23	4.4%			
ER							
Neg	10	34.5%	101	19.3%	0.048	2.19	0.99-4.86
Pos	19	65.5%	421	80.7%			
PR							
Neg	13	44.8%	153	29.4%	0.078	1.95	0.92-4.16
Pos	16	55.2%	368	70.6%			
HER2							
Neg	24	82.8%	447	85.6%	0.595	0.81	0.30-2.18
Pos	5	17.2%	75	14.4%			
Grade							
1	4	12.9%	86	17.1%			
2	14	45.2%	226	44.9%	0.659	0.85	0.41-1.77
3	13	41.9%	191	38.0%	1 and 2 vs. 3		
Tumor histology							
Ductal	23	79.2%	395	75.7%			
Lobular	3	10.4%	90	17.2%	0.656	1.23	0.49-3.09
Medullary	0	0%	2	0.4%	Ductal vs. all other		
Other	3	10.4%	35	6.7%			
Type							
LumA	16	55.2%	385	73.8%			
LumB	3	10.3%	43	8.2%	0.021	2.83	1.20-6.66
HER2	2	6.9%	32	6.1%	Triple-neg vs. other		
Triple-neg	8	27.6%	62	11.9%			
Ki67							
0	1	3.6%	71	13.7%			
1	13	46.4%	231	44.5%	0.393	0.72	0.34-1.54
2	7	25.0%	112	21.6%	0 and 1 vs. 2 and 3		
3	7	25.0%	105	20.2%			

T = tumor size, N = nodal status, M = primary metastasis, ER = estrogen receptor, PR = progesterone receptor, LumA = luminalA, LumB = luminalB, Neg = negative, Pos = positive.

^a^not corrected for multiple testing.

## Discussion

Inherited genomic alterations are expected to have an effect on individual’s risk of getting cancer. However, these alterations may affect not only the risk but also the pattern of somatically acquired mutations and thereby tumor biology. Much of the work in this field has been concentrating on single nucleotide polymorphisms (SNPs) whereas the role of CNVs has remained poorly defined, partially because their detection with Sanger or even with sophisticated Next-Generation sequencing is hard or even impossible, and the fast and cost-efficient investigation of CNV alleles requires the characterization of their breakpoints in exact detail. Of the currently studied rare CNV alleles, the breakpoints of five out of six were adequately well described by the used analysis software, indicating that high-resolution microarrays can predict fairly well the genuine genomic coordinates of the aberrations. The singleton CNVs previously identified in familial or early-onset cases [4] were absent from, or remained extremely rare in the unselected breast cancer cases, but the two recurrent alleles were observed at higher frequency. The carrier frequency of the *CDH19* deletion was twice as high in unselected breast cancer cases as in controls but remained below statistical significance, indicating the need for larger case–control cohorts to demonstrate its association with breast cancer. The other recurrent CNV allele, *CYP2C19* deletion, had surprisingly high prevalence both in the studied cases and controls (>3%), particularly when considering that deletion CNVs have rarely been reported in CYP genes, despite the numerous studies performed. This can be explained by the poor detection of CNV alleles by conventional genotyping methods, but also by the fact that our study was performed with samples from Finnish founder population. Founder populations are known to harbor unique mutations and some of the mutations rare in other populations can also show enrichment in them. In regard to cancer predisposition, the effect of the *CYP2C19* deletion was reminiscent to that of a low-penetrance allele. However, it was found to be associated specifically with the triple-negative molecular subtype of the breast cancer.

There are multiple lines of evidence for the profound role of estrogen in breast cancer development: disruptions in estrogen signalling and metabolism have long been considered to affect breast cancer risk. This can result from different reproductive and hormonal factors [25], but could also be due to variations in the enzymatic machinery responsible for estrogen metabolism. Indeed, the currently studied *CYP2C19* deletion CNV is expected to result in a null allele of a gene encoding an enzyme involved in estrogen catabolism [5, 6]. All currently identified mutation positive individuals were heterozygous for the *CYP2C19* deletion, and the genotype of the remaining allele seemed not to play a role in the observed association with tumor triple-negativity. This could be explained by a genuine haploinsufficient situation, in which single allele is unable to sustain full functionality when compared to the protein levels produced by two wild type alleles. Alternatively, as the *CYP2C19* deletion allele extends over 60 kb, starting only 1377 bp from the 3’ end of the adjacent *CYP2C18* gene, it could change the genomic landscape of this region in a way that leads to aberrant expression of both genes. It is also possible that large genomic deletions disturb the communication between the homologous alleles required for their full function by deleting regulatory elements required for this process [26].

Another *CYP2C19* allele, CYP2C19*17, defining an ultra-rapid metabolizer phenotype, has previously been associated with a decreased risk for breast cancer. This suggests that increased catabolism of estrogens by CYP2C19 may lead to decreased estrogen levels and therefore reduced breast cancer risk [27]. Correspondingly, our initial hypothesis was that *CYP2C19* deletion allele effects are mediated through life-long increased estrogen levels. Why this would predispose particularly to ER negative breast cancer is currently, however, puzzling and the mechanism through which the *CYP2C19* deletion operates remains unclear. However, any perturbations in estrogen metabolism are still among the possible explanations. Curiously, there are also reports linking obesity with triple-negative breast cancer [28]. Although obesity-related insulin resistance and chronic inflammatory could be possible explanations for this phenomenon, increased body mass index is known to cause changes in the hormonal cycles and result in excessive adipose tissue [29, 30]. This can increase the estrogen production and availability, leading again to the unexpected association with receptor negative breast cancer. Nevertheless, as triple-negative breast cancer is a tumor subtype with unique characteristics not only in its pathological presentation but also in prognosis and response to therapy, identification of additional risk factors specifically associated with this subgroup of breast cancer could help to understand its etiology.

## Conclusion

Our results indicate that an inherited defect in the *CYP2C19* gene with a role in estrogen catabolism has an influence on the molecular subtype of breast cancer and is significantly associated with triple-negative tumors. The role of the *CYP2C19* deletion allele, as well as that of the *CDH19* deletion, in breast cancer predisposition warrants further studies and the obtained results should be replicated with larger and independent case–control cohorts.

## Electronic supplementary material

Additional file 1: Table S1: Primers used in multiplex PCR. **Table S2.** Primers used for the detection of other metabolizer phenotypes described in CYP2C19. **Table S3.** Correspondence of microarray based and sequencing confirmed genomic coordinates for the breakpoints. **Table S4.** Tumor characteristics of *CDH19* deletion allele carriers compared with the tumors of non-carrier unselected breast cancer cases. (DOCX 28 KB)

Additional file 2: Figure S1: Sequence of the *CYP2C19* deletion breakpoint. (PPTX 321 KB)
