# Supplementary material for: Recurrent CYP2C19 deletion allele is associated with triple-negative breast cancer
Source: BMC Cancer. 2014 Dec 2;14:902. doi: 10.1186/1471-2407-14-902 (PMC4265448; doi:10.1186/1471-2407-14-902)
Supplement: Supplementary file 1 — Additional file 1: Table S1: Primers used in multiplex PCR. Table S2. Primers used for the detection of other metabolizer phenotypes described in CYP2C19. Table S3. Correspondence of microarray based and sequencing confirmed genomic coordinates for the breakpoints. Table S4. Tumor characteristics of CDH19 deletion allele carriers compared with the tumors of non-carrier unselected breast cancer cases. (DOCX 28 KB) [file 12885_2014_5069_MOESM1_ESM.docx]

**Table S1.** Primers used in multiplex PCR.

| **Primer** | **Sequence 5' - 3'** |
| --- | --- |
| CYP2C19F | AAGCCATGAAGCCATCAACT |
| CYP2C19R | AAAGTGTTAAGGGCAGCAGAG |
| CDH19F | CCTAGGCAATTCCACTCAGAA |
| CDH19R | CAGGATTTCAAAAAGCACACA |
| DCLRE1CF | gtggctcactagagcccagc |
| DCLRE1CR | GCACAGTCACGGCTCACTATAA |
| ITGA9F | CCCAGTGGTTCTCAACCAAG |
| ITGA9R | GCTTTGAAGTAGGAGAATGTGTGA |
| CASP3F | CCATCGCTCACATCATTACCT |
| CASP3R | CCCGAGAGTTCGGAGATACC |
| DAB2IPF | CACAGCCCTTAAGACCAGTCA |
| DAB2IPR | CTGGCTGGAGGGAACGTC |
| PALB2F (PCR control) | ACAGCGCGGCTCTCCTTTAG |
| PALB2R (PCR control) | ATACTGCTGCCCTCGGACTG |

F = forward primer

R = reverse primer

**Table S2.** Primers used for the detection of other metabolizer phenotypes described in *CYP2C19.*

| ***CYP2C19* variant** | **Metabolizer phenotype** | **Forward 5’-3’** | **Reverse 5’-3’** |
| --- | --- | --- | --- |
| *2 (c.681G>A, rs4244285) | poor | AATTACAACCAGAGCTTGGC | GTAAACACAAAACTAGTCAATG |
| *3 (c.636G>A, rs57081121) | poor | CCAATCATTTAGCTTCACCC | ACTTCAGGGCTTGGTCAATA |
| *17 (‑806C>T, rs12248560) | ultra-rapid | GCCCTTAGCACCAAATTCTC | ATTTAACCCCCTAAAAAAACACG |

**Table S3.** Correspondence of microarray based and sequencing confirmed genomic coordinates for the breakpoints.

| **Disrupted** |  | **Predicted by array^1^** | | |  | **Sequencing confirmed** | | |
| --- | --- | --- | --- | --- | --- | --- | --- | --- |
| **gene** | **Chr** | **Start** | **End** | **Size (bp)** |  | **Start** | **End** | **Size (bp)** |
| *CYP2C19* | 10 | 96,497,371 | 96,558,977 | 61607 |  | 96,497,324 | 96,559,110 | 61786 |
| *CDH19* | 18 | 64,081,296 | 64,339,529 | 258234 |  | 64,082,045 | 64,335,669 | 253624 |
| *ITGA9* | 3 | 37,747,296 | 37,807,058 | 59763 |  | 37,750,166 | 37,810,925 | 60759 |
| *CASP3* | 4 | 185,507,794 | 185,840,382 | 332589 |  | 185,506,876 | 185,841,468 | 334592 |
| *DAB2IP* | 9 | 124,240,146 | 124,344,069 | 103924 |  | 124,201,774 | 124,361,084 | 159310 |
| *DCLRE1C* | 10 | 14,986,306 | 15,065,749 | 79444 |  | 14,983,925 | 15,065,676 | 81751 |

^1^ Breakpoint coordinates were received through analysis with GenomeStudio Genotyping module (Illumina) and Nexus Copy Number Discovery Edition 5.1 software (BioDiscovery Inc.). Genomic coordinates according to human genome assembly 19 (February 2009).

**Table S4.** Tumor characteristics of *CDH19* deletion allele carriers compared with the tumors of non-carrier unselected breast cancer cases.

| **Category** | ***CDH19***  **deletion** | **%** | **WT** | **%** | ***P*-value ^a^** | **OR** | **95%** |
| --- | --- | --- | --- | --- | --- | --- | --- |
| T |  |  |  |  |  |  |  |
| 1 | 7 | 58.3% | 315 | 58.6% |  |  |  |
| 2 | 5 | 41.7% | 193 | 35.9% | 1.00 | 0.99 | 0.31-3.16 |
| 3 | 0 | 0% | 17 | 3.1% | 1 vs. 2,3,4 |  |  |
| 4 | 0 | 0% | 13 | 2.4% |  |  |  |
| N |  |  |  |  |  |  |  |
| Neg | 7 | 58.3% | 306 | 56.5% | 0.897 | 1.08 | 0.34-3.45 |
| Pos | 5 | 41.7% | 236 | 43.5% |  |  |  |
| M |  |  |  |  |  |  |  |
| Neg | 12 | 100% | 519 | 95.8% | 1.00 | 1.04 | 1.03-1.06 |
| Pos | 0 | 0% | 23 | 4.2% |  |  |  |
| ER |  |  |  |  |  |  |  |
| Neg | 2 | 18.2% | 109 | 20.2% | 1.00 | 0.88 | 0.19-4.13 |
| Pos | 9 | 81.8% | 431 | 79.8% |  |  |  |
| PR |  |  |  |  |  |  |  |
| Neg | 6 | 54.5% | 160 | 29.7% | 0.097 | 2.84 | 0.86-9.45 |
| Pos | 5 | 45.5% | 379 | 70.3% |  |  |  |
| HER2 |  |  |  |  |  |  |  |
| Neg | 8 | 72.7% | 463 | 85.7% | 0.205 | 0.44 | 0.12-1.71 |
| Pos | 3 | 27.3% | 77 | 14.3% |  |  |  |
| Grade |  |  |  |  |  |  |  |
| 1 | 1 | 8.3% | 89 | 17.1% |  |  |  |
| 2 | 4 | 33.3% | 236 | 45.2% | 0.227 | 0.43 | 0.14-1.38 |
| 3 | 7 | 58.3% | 197 | 37.7% | 1 and 2 vs. 3 |  |  |
| Tumor histology |  |  |  |  |  |  |  |
| Ductal | 11 | 100% | 407 | 75.4% |  |  |  |
| Lobular | 0 | 0% | 93 | 17.2% | 0.074 | 1.33 | 1.26-1.39 |
| Medullary | 0 | 0% | 2 | 0.4% | Ductal vs. all other |  |  |
| Other | 0 | 0% | 38 | 7.0% |  |  |  |
| Type |  |  |  |  |  |  |  |
| LumA | 7 | 63.6% | 394 | 73.0% |  |  |  |
| LumB | 2 | 18.2% | 44 | 8.1% |  |  |  |
| HER2 | 1 | 9.1% | 33 | 6.1% | 1.00 | 0.68 | 0.09-5.42 |
| Triple-neg | 1 | 9.1% | 69 | 12.8% | triple-neg vs. all other |  |  |
| Ki67 |  |  |  |  |  |  |  |
| 0 | 1 | 9.1% | 71 | 13.2% |  |  |  |
| 1 | 4 | 36.3% | 240 | 44.8% | 0.54 | 0.6 | 0.18-2.00 |
| 2 | 3 | 27.3% | 116 | 21.7% | 0 and 1 vs. 2 and 3 |  |  |
| 3 | 3 | 27.3% | 109 | 20.3% |  |  |  |

T = tumor size, N = nodal status, M = primary metastasis, ER = estrogen receptor, PR = progesterone receptor, LumA = luminalA, LumB = luminalB, Neg = negative, Pos = positive.

**^a^** not corrected for multiple testing.
