# Supplementary figures and images for: Recurrent CYP2C19 deletion allele is associated with triple-negative breast cancer
Source: BMC Cancer. 2014 Dec 2;14:902. doi: 10.1186/1471-2407-14-902 (PMC4265448; doi:10.1186/1471-2407-14-902)

## Slide 1
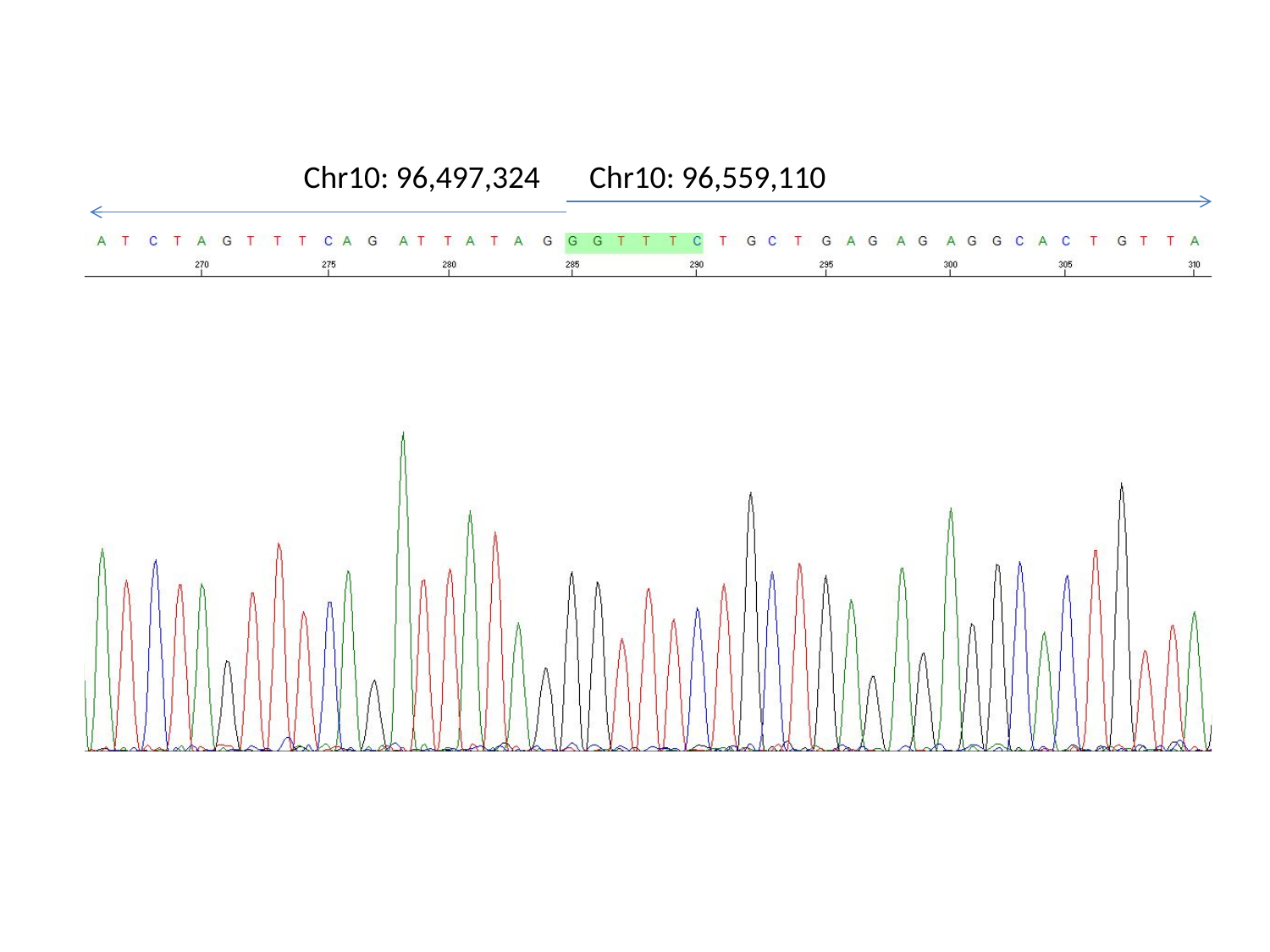

Chr10: 96,497,324
Chr10: 96,559,110

Supplement: Supplementary file 2 — Additional file 2: Figure S1: Sequence of the CYP2C19 deletion breakpoint. (PPTX 321 KB) [file 12885_2014_5069_MOESM2_ESM.pptx]
